# Herpes Zoster Meningitis Complicating Combined Tocilizumab and Cyclosporine Therapy for Adult-Onset Still's Disease

**DOI:** 10.1155/2016/4232657

**Published:** 2016-03-22

**Authors:** Shinichiro Tsurukawa, Nozomi Iwanaga, Yasumori Izumi, Atsunori Shirakawa, Chieko Kawahara, Tetsuo Shukuwa, Miwako Inamoto, Atsushi Kawakami, Kiyoshi Migita

**Affiliations:** ^1^Department of Rheumatology, Clinical Research Center, NHO Nagasaki Medical Center, Kubara 2-1001-1, Omura 856-8562, Japan; ^2^Department of Pharmacy, Clinical Research Center, NHO Nagasaki Medical Center, Kubara 2-1001-1, Omura 856-8562, Japan; ^3^Department of Dermatology, Clinical Research Center, NHO Nagasaki Medical Center, Kubara 2-1001-1, Omura 856-8562, Japan; ^4^Department of Ophthalmology, Clinical Research Center, NHO Nagasaki Medical Center, Kubara 2-1001-1, Omura 856-8562, Japan; ^5^Department of Immunology and Rheumatology, Unit of Translational Medicine, Nagasaki University Graduate School of Biomedical Sciences, Sakamoto 1-7-1, Nagasaki 852-8501, Japan

## Abstract

A 56-year-old female with refractory adult-onset Still's disease presented with ocular herpes zoster infection during TCZ treatment. After three days of acyclovir treatment (5 mg/kg), she developed a severe headache and high fever. Viral DNA isolation and cerebral spinal fluid abnormalities led to a herpes zoster meningitis diagnosis. Her meningitis was cured by high doses of intravenous acyclovir (10 mg/kg for 14 days). To our knowledge, this is the first report of meningeal herpes zoster infection in rheumatic diseases under TCZ treatment.

## 1. Introduction 

Opportunistic viral infections are a well-recognized complication of biologics treatments for rheumatic diseases [1]. Cases of herpes zoster infections have been described in association with the immunosuppression induced by some rheumatic disease treatments [2]. Herpes zoster virus not only causes a primary infection (varicella/chickenpox) but also can be reactivated from a latent state, in which it is sequestered in the dorsal root ganglia [3]. A large prospective cohort of 3,266 rheumatoid arthritis (RA) patients on anti-TNF therapy found an increased risk of herpes zoster infection in RA patients, and the severity of many of these cases required hospitalization [4]. However, central nervous system herpes zoster viral infections have rarely been described in patients receiving TNF inhibitors [5], and, to our knowledge, there are no previous reports of herpes zoster meningitis associated with anti-IL-6 therapy. Here, we present the case of a 56-year-old female with adult onset Still's disease (AOSD) who developed herpes zoster meningitis while being treated with cyclosporin A, prednisolone, and an anti-IL-6 therapeutic, tocilizumab (TCZ).

## 2. Case Report

A 56-year-old female was referred to our hospital with a three-day history of left eye corneitis and a left frontal headache. She had been diagnosed with a systemic type of AOSD in 1999. Initial treatment with immunosuppressants (MTX and leflunomide) and biologics (TNF inhibitors and abatacept), which were combined with steroid treatments, had to be discontinued because of insufficient effects.

The patient had steroid-refractory AOSD; so, from 2010, she had been treated with TCZ (8 mg/kg in 4-week intervals) and cyclosporin A (200 ng/day) in addition to steroid therapy. The systemic manifestations of AOSD, such as spiking fever, skin rash, and hyperferritinemia, were controlled by these treatments. A herpes zoster infection occurred five days after one of the TCZ infusions. At admission, the patient was afebrile and had a unilateral vesicular eruption with a well-defined dermatomal distribution involving the left ophthalmic verve area (trigeminal nerve V1) with corneal erosion but without visceral involvement. An acyclovir infusion treatment (5 mg/kg) was promptly started.

Three days after admission, the patient presented with high fever, severe headache, and vomiting. A detailed neurological examination demonstrated no focal motor or sensory deficits, and the cranial nerve test results were normal. A fundoscopy did not reveal any papilledema. Additionally, there was no nuchal rigidity, and both Brudzinski's sign and Kernig's sign were negative.

Laboratory studies revealed a peripheral white blood cell count of 9,600 cells/*μ*L, with 77% neutrophils, 13% lymphocytes, and 5% monocytes. C-reactive protein was not detected, but the patient's liver transaminases and serum ferritin were slightly elevated (Table 1). A MRI examination of the patient's head was unremarkable. A lumbar puncture was performed, and the resulting cerebrospinal fluid (CSF) revealed an elevated protein level (92 mg/dL), decreased glucose level (45 mg/dL with a blood sugar level of 96 mg/dL), and a marked pleocytosis (55 cells/*μ*L with 91% monocytes). While awaiting the results of a herpes zoster DNA detection test, we began treatment with a high dose of intravenous acyclovir (10 mg/kg) for 14 days based on a suspicion of herpes zoster meningitis. Later, a polymerase chain reaction assay of the CSF detected herpes zoster viral DNA, confirming our suspicions (Table 1). The patient's symptoms were improved by the high-dose acyclovir treatments, and she was discharged from the hospital after a total stay of 20 days. The elevated serum levels of transaminases and ferritin were also normalized by this therapy.

## 3. Discussion

To our knowledge, this is the first reported case of herpes zoster meningitis in an AOSD patient taking TCZ, and it highlights the risk of atypical and severe herpes zoster infection among immunosuppressed patients receiving biologics. In our case, the ophthalmic herpes zoster infection disseminated to a meningeal infection. As disseminated herpes zoster infections cause sequelae of the nervous system, early viral detection, antiviral therapy, and prevention strategies are particularly important for these patients [6].

The herpes zoster infection risk in rheumatic disease patients is high, and this risk appears to be increased in patients treated with biologics [2]. For example, in the SAMURAI study [7], the incidence of serious infections was 7.6% (0.6% herpes zoster) in the TCZ group versus 4.1% in the DMARDs group. Similarly, in the TOWARD study [8], the rate of serious infections per 100 patient-years was 5.9 (0.4% herpes zoster) in the TCZ group versus 4.7 in the control group. Likewise, the extension STREAM study [9] found a rate of serious infection of 5.7 per 100 patient-years and a rate of serious herpes infections of 1.1 events per 100 patient-years. The published rates of herpes zoster incidence during TCZ treatment are similar to those during treatment with anti-TNF inhibitors [4].

This case of herpes zoster meningitis in a patient with AOSD who was treated with TCZ must be considered within the context of herpes zoster in immunocompromised patients. In addition to the TCZ treatment, the patient was also treated with other immunosuppressive therapies, including cyclosporin A and steroids. Therefore, the herpes zoster infection risk attributable to the anti-IL-6 agent in this case is confounded by the immunosuppression caused by the combined treatment with prednisolone and cyclosporin A. Notably, other studies have also reported a higher risk for herpes zoster viral infection among patients receiving combination of immunosuppressive therapies. Specifically, glucocorticoid use had a significant association with herpes zoster, so it is likely that the usage of a moderate dose of glucocorticoid, in addition to cyclosporin A, could have contributed to the occurrence of herpes zoster meningitis seen in the present case.

Herpes zoster is caused by the reactivation of latent varicella zoster virus (VZV) maintained in the spinal dorsal root ganglia of the cranial nerves after primary infection. Virus replication following the primary infection depends upon several conditions, and protection from VZV reactivation from latency seems to depend on the VZV-specific T cell-mediated immunity that is elicited during the primary infection and declines with advancing age [10]. Meningoencephalitis, a rare manifestation of herpes zoster virus reactivation presumably caused by a centripetal spread of virus along the posterior root of the spinal cord, has been described in immunocompetent patients [11]. Other reports have demonstrated that IL-6 is required for an optimal immune response after ocular herpes simplex virus type 1 infection and is considered to contribute to resistance against herpesviruses [12].

Herpes zoster infections can be associated with subtle signs of aseptic meningitis, such as mild CSF mononuclear pleocytosis with a slight increase in protein, in up to 50% of patients [13], but this is rarely accompanied by virus isolation from the CSF. Therefore, when herpes zoster virus can be implicated in CNS infections in immunocompetent patients, investigations of the CSF are mandatory. For laboratory confirmation of herpes zoster virus, viral DNA detection by PCR, as was used in this case, is much more sensitive than virus culture, which has a low yield. Based on case reports and small series [14], a guideline from the Infectious Diseases Society of America suggests treating VZV encephalitis with intravenous acyclovir at 10–15 mg/kg every 8 hours for 10–14 days. Therefore, we treated the patient with 10 mg/kg for 14 days [15].

Immunization with a live attenuated vaccine reduces the risk of recurrent herpes zoster infection among immunocompetent individuals [16]. According to the American College of Rheumatology, however, the live herpes zoster vaccine is contraindicated in patients receiving immunosuppressive medications, such as biologic agents. Surprisingly [17], a recent study indicated that herpes zoster vaccination was not associated with a short-term increase in the herpes zoster incidence of Medicare beneficiaries with immune-mediated diseases, including those receiving biologics [18]. These data call into question the current recommendation of herpes zoster vaccine contraindication for patients receiving biologics. Therefore, further studies concerning the safety and effectiveness of herpes zoster vaccination among patients receiving biologics are needed.

In conclusion, this paper presents the first reported case of herpes zoster meningitis occurring opportunistically in association with TCZ therapy for AOSD. This case highlights the risk of severe, atypical opportunistic infections and the need for both early recognition of herpes zoster meningitis and aggressive management of this disease with antiviral therapy. It also draws attention to the potential cofounders of concomitant immunosuppression in the course of AOSD treatments, including TCZ.

## Figures and Tables

**Table 1:** Laboratory findings.

Peripheral blood		Serological tests	
Red blood cells	406 × 10^4^/*μ*L	C-reactive protein	<0.30 mg/dL
Hemoglobin	10.5 g/dL	Erythrocyte sedimentation rate	3.0 mm/hr
White blood cells	9600/*μ*L	IgG	883 mg/dL (900–2000)
St	23.0%	Ferritin	613 ng/mL (10–85)
Seg	66.0%	C3	108 mg/dL (4–64)
Ly	6.0%	C4	24 mg/dL (17–45)
Mo	4.0%	CH50	42.7 U/mL (30–50)
AtyLy	1.0%	Anti-nuclear Ab	<×40
Platelet	12.1 × 10^4^/*μ*L	Anti-CCP Ab	<0.6 U/mL (<4.5)
Blood chemistry		*β*-D-glucan	(−)
Total protein	5.9 g/dL		
Albumin	3.9 g/dL	CSF	
Total bilirubin	0.6 mg/dL	Cell number	55/mm^3^
Glutamic-oxaloacetic transaminase	176 IU/L (7–33)	Neutrophils	3%
Glutamic-pyruvic transaminase	181 IU/L (5–30)	Monocytes	97%
Lactate dehydrogenase	518 IU/L (260–480)	****	
Alkaline phosphatase	271 IU/L (80–250)	Protein	92 mg/dL
Creatinine kinase	21 IU/L (60–160)	Sugar	45 mg/dL
Total cholesterol	217 mg/dL	Cl	103 mEq/L
Blood urea nitrogen	10.8 mg/dL	Herpes zoster DNA (PCR method)	(+)
Creatinine	0.4 mg/dL		
Na	128 mEq/L		
K	3.5 mEq/L		
Cl	95 mEq/L		

Anti-CCP Ab, anti-cyclic citrullinated peptide antibody; CSF, cerebrospinal fluid; PCR, polymerase chain reaction.
